# The Receptor AT1 Appears to Be Important for the Maintenance of Bone Mass and AT2 Receptor Function in Periodontal Bone Loss Appears to Be Regulated by AT1 Receptor

**DOI:** 10.3390/ijms222312849

**Published:** 2021-11-27

**Authors:** Maria Laura de Souza Lima, Agnes Andrade Martins, Caroline Addison Carvalho Xavier de Medeiros, Gerlane Coelho Bernardo Guerra, Robson Santos, Michael Bader, Flavia Q. Pirih, Raimundo Fernandes de Araújo Júnior, Gerly Anne de Castro Brito, Renata Ferreira de Carvalho Leitão, Rafaela Alcindo Silva, Stphannie Jamyla de Araújo Barbosa, Rômulo Camilo de Oliveira Melo, Aurigena Antunes de Araújo

**Affiliations:** 1 Postgraduate Program in Dentistry Sciences, Department of Biophysical and Pharmacology, Federal University of Rio Grande Norte, Natal CEP 59072-970, RN, Brazil; mlauradesouzalima@gmail.com; 2 Department of Dentistry, Federal University of Rio Grande Norte, Natal CEP 59072-970, RN, Brazil; agnesandrade7@gmail.com; 3 Postgraduate Program in Biochemistry and Molecular Biology, Postgraduate Program in RENORBIO, Department of Biophysical and Pharmacology, Federal University of Rio Grande Norte, Natal CEP 59072-970, RN, Brazil; carolaufrn@gmail.com; 4 Postgraduate Program in Biochemistry and Molecular Biology, Postgraduate Program in Pharmaceutical Science, Department of Biophysical and Pharmacology, Federal University of Rio Grande Norte, Natal CEP 59072-970, RN, Brazil; gerlaneguerra@hotmail.com; 5 Department of Physiology, Federal University of Minas Gerais, Belo Horizonte CEP 31270-901, MG, Brazil; robsonsant@gmail.com; 6 Max Delbrück Center for Molecular Medicine, 13125 Berlin, Germany; mbader@mdc-berlin.de; 7 School of Dentistry, University of California-Los Angeles (UCLA), Los Angeles, CA 90095, USA; fpirih@dentistry.ucla.edu; 8 Postgraduate Program in Health Sciences, Postgraduate Program in Functional and Structural Biology UFRN, Department of Morphology, Federal University of Rio Grande Norte, Natal CEP 59072-970, RN, Brazil; araujojr.morfologia@gmail.com; 9 Postgraduate Program in Morphofunctional Sciences, Department of Morphology, School of Medicine, Federal University of Ceará, Fortaleza CEP 60430-170, CE, Brazil; gerlybrito@hotmail.com; 10 Postgraduate Program in Morphology, Department of Morphology, Federal University of Ceará, Fortaleza CEP 60430-170, CE, Brazil; leitao_renata@yahoo.com.br; 11 Postgraduate Program in Dentistry Sciences, Department of Dentistry, Federal University of Rio Grande Norte, Natal CEP 59072-970, RN, Brazil; rafaela.alciindo@hotmail.com; 12 Postgraduate Program in Pharmaceutical Science, Department of Pharmaceutical Science, Federal University of Rio Grande Norte, Natal CEP 59072-970, RN, Brazil; stpfarma@gmail.com; 13 Discipline of Radiology, Department of Dentistry, Federal University of Rio Grande Norte, Natal CEP 59072-970, RN, Brazil; camilo-melo@hotmail.com; 14 Visiting Research in UCLA, Postgraduate Program in Dentistry Sciences, Postgraduate Program in Pharmaceutical Sciences, Department of Biophysical and Pharmacology, Federal University of Rio Grande Norte, Natal CEP 59072-970, RN, Brazil

**Keywords:** bone, micro-computed tomography, periodontal disease, molecular

## Abstract

A large number of experimental studies has demonstrated that angiotensin II (Ang II) is involved in key events of the inflammatory process. This study aimed to evaluate the role of Ang II type 1 (AT1) and Ang II type 2 (AT2) receptors on periodontitis. **Methods:** Experimental periodontitis was induced by placing a 5.0 nylon thread ligature around the second upper left molar of AT1 mice, no-ligature or ligature (AT1-NL and AT1-L), AT2 (AT2-NL or AT2-L) and wild type (WT-NL or L). Alveolar bone loss was scanned using Micro-CT. Cytokines, peptides and enzymes were analyzed from gingival tissues by Elisa and RT-PCR. **Results:** The blockade of AT1 receptor resulted in bone loss, even in healthy animals. Ang II receptor blockades did not prevent linear bone loss. Ang II and Ang 1-7 levels were significantly increased in the AT2-L (*p* < 0.01) group compared to AT2-NL and AT1-L. The genic expression of the Mas receptor was significantly increased in WT-L and AT2-L compared to (WT-NL and AT2-NL, respectively) and in AT1-L. **Conclusions:** Our data suggest that the receptor AT1 appears to be important for the maintenance of bone mass. AT2 receptor molecular function in periodontitis appears to be regulated by AT1.

## 1. Introduction

Periodontitis is a chronic inflammatory, multicausal disease [1] and a leading cause of tooth loss in adults [2].

The association between periodontitis and cardiovascular disease is proven, and this relationship appears to be independent of other classical risk factors [3]. A critical role as a mediator between the two diseases seems to be inflammation.

The control of electrolyte homeostasis and blood pressure in the body is performed by the renin-angiotensin system (RAS). In addition to this vital function, it is also responsible for modulating some biological functions such as oxidative stress, inflammation, and hormone peptide release [4,5,6].

On top of the systemic presence of the RAS, there is the presence of a tissue-independent subsystem which acts in an autocrine and paracrine manner, and an intracellular system [7,8,9]. RAS components have been proven to be present in various tissues, such as the kidneys, ovaries, liver, brain, adipose tissue, in addition to its presence in oral tissues [8,9,10].

The classic cascade of the RAS starts from the cleavage of Angiotensinogen (AGT) into Angiotensin I (Ang I) by the action of Renin. Next, Ang I is converted to Angiotensin II (Ang II) by Angiotensin-converting enzyme (ACE). Angiotensin II can act or be converted to Angiotensin 1-7 (Ang 1-7) by Angiotensin-converting enzyme 2 (ACE2). Ang I can also be cleaved into Angiotensin 1-7 (Ang 1-9) by the action of ACE2, which can also generate Ang 1-7 [6,8,11]. Angiotensin 1-7 interacts with the Mas-type receptor (MasR) which, like AT2R, is considered part of the protective arm of the renin-angiotensin system [12,13].

Angiotensin II is the main molecule of RAS and mainly interacts with two G-protein-coupled receptors, angiotensin II type 1 (AT1) and angiotensin II type 2 (AT2) receptors [5]. The interaction with type I receptors promotes the classic effects of angiotensin II such as vasoconstriction, inflammation, oxidative stress, increased proliferation factors, and cardiovascular effects, among others [6,14]. This angiotensin II receptor is shown to be an essential pathophysiological activator of inflammatory mechanisms [15]. On the other hand, the interaction with the less expressed type 2 receptor is associated with protective effects [11] and counter regulates AT1 actions, while AT1 is responsible for AT2 regulation [14].

The binding of Angiotensin II to the AT1 receptor increases vascular permeability and the production of vascular endothelial growth factor (VEGF), in addition to stimulating the L-selectin expressed by neutrophils and adhesion molecules expressed by endothelial cells [4,16]. Activation of this receptor also induces leukocyte migration, release of pro-inflammatory cytokines, such as tumor necrosis factor alpha (TNF-α), IL-1β, IL-6, and NF-κB activation [4,12]. Activation of the AT2 receptor has been shown to inhibit the production of pro-inflammatory cytokines and the recruitment of T cells and to decrease the production of superoxide [17].

Vascular diseases are nowadays recognized as having a strong local and systemic inflammatory pathogenic contribution. Periodontitis and its chronic inflammatory nature are associated with an increased risk of cardiovascular disease [18]. The inflammatory response associated with cardiovascular disease and also chronic periodontitis is modulated by proinflammatory cytokines such as TNF-α, IL-1, and IL-6 [19].

A significant increase in the gene expression of ACE, AT1 receptors and inflammatory mediators, such as proinflammatory cytokines, has been reported in ligature-induced periodontitis [4]. It has been suggested that Angiotensin II type I receptors (AT1) modulate the progression of experimental periodontitis, since treatment of rats undergoing ligature-induced periodontitis with Losartan or Telmisartan, both antagonists of AT1 receptors, has protective effects on the progression of periodontitis. The aim of the present work, therefore, was to investigate the participation of both AT1 and AT2 receptors in experimental periodontitis through the constitutive silencing of these receptors. As far as we know, this study is the first to submit AT1 and AT2 knockout mice to ligature-induced periodontitis.

## 2. Results

### 2.1. Analysis of Linear Bone Loss and Bone Volume by Micro-Computed Tomography (Micro-CT)

**Figure 1:**
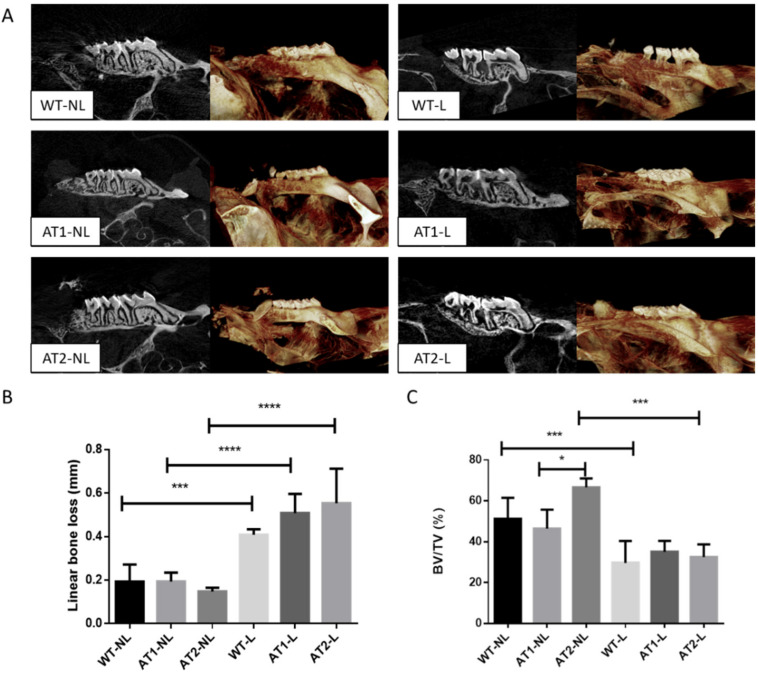
**Micro-CT.** (**A**) 3D reconstruction and 2D image of the maxilla of healthy and ligation-induced periodontal disease animals; (**B**) linear bone loss results; (**C**) bone volume ratio results. Mean and standard deviation. Statistical differences by brackets labeled by * *p* < 0.05, *** *p* < 0.001, **** *p* < 0.0001 comparing WT-NL vs. WT-L, AT1-NL vs. AT1-L, AT2-NL vs. AT2-L, AT1-NL vs. AT2-NL, AT1-L vs. AT2-L, WT-L vs. AT1-L. (Graph Pad Prism 6.01 software). *n* = 5 animal per group.

The standard clinical feature for diagnosing periodontitis is bone loss and periodontal pocket formation. Thus, a crucial analysis for understanding the severity of the disease is through the linear bone loss and bone volume ratio parameters. These data can be visualized through two-dimensional (2D) and three-dimensional (3D) images performed through the micro-CT (Figure 1A).

The linear bone loss results (Figure 1B) showed an increase in the distance from the cement–enamel junction in animals submitted to ligature-induced periodontitis when compared to their respective control groups (*p* < 0.001), confirming that ligature was able to induce bone loss, a hallmark of periodontitis progression.

The ratio of bone volume (BV) to total volume (TV) (BV/TV%) showed that bone loss was significantly higher in WT-L and AT2-L groups when compared to their respective controls, WT-NL and AT2-NL (*p* < 0.001). No significant difference was found between AT1-L and AT1-NL. A smaller bone volume was found in the AT1-NL group when compared to AT2-NL (*p* < 0.05), suggesting that AT1 receptors appear to be important for the maintenance of bone mass.

### 2.2. Analysis of Cytokines and Peptides by ELISA Immunoassay

**Figure 2:**
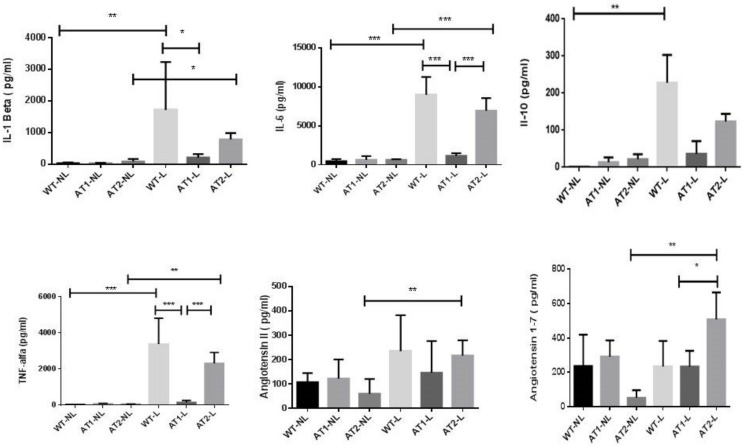
**IL-1, IL-6, IL-10, TNF-, Ang 1-7 and Ang II levels for the no-ligature and ligature groups of the three knockout mouse strains by enzyme-linked immunosorbent assay (ELISA).** Dosage of IL-1 levels; Dosage of IL-6 levels; Dosage of Il-10 levels; Dosage of TNF-levels; Dosage of Ang 1-7 levels; Dosage of Ang II levels. All graphs show mean and standard deviation (*n* = 5/group). Statistical differences by brackets labeled by * *p* < 0.05, ** *p* < 0.01, *** *p* < 0.001, comparing WT-NL vs. WT-L, AT1-NL vs. AT1-L, AT2-NL vs. AT2-L, AT1-L vs. AT2-L, WT-L vs. AT1-L. (Graph Pad Prism 6.01 software).

Thus, our group evaluated IL-1β, IL-6 and TNF-α pro-inflammatory cytokine levels as well as the levels of the anti-inflammatory cytokine IL-10. Both WT-L and AT2-L groups of animals submitted to ligature-induced periodontitis showed a significant increase in IL-1β level (Figure 2) when compared to their respective control groups, without ligature, WT-NL and AT2-NL (*p* < 0.01 and *p* < 0.05, respectively). AT1-L knockout mice submitted to ligature-induced periodontitis (AT1-L group) and had a significantly lower level of IL-1β in gingival tissue compared to wild type animals (WT-L) (*p* < 0.05).

Both WT-L and AT2-L groups had higher levels of IL-6 (Figure 2) compared to their control groups, WT-NL and AT2-NL, respectively (*p* < 0.001). AT1-L knockout mice submitted to ligature-induced periodontitis (AT1-L group) and had a significantly lower level of IL-6 in gingival tissue when compared to wild type animals (WT-L) and AT2 knockout mice, both submitted to ligature-induced periodontitis (*p* < 0.001).

The levels of IL-10 were higher in the WT-L group (*p* < 0.01) compared to its control group, WT-NL (Figure 2).

Both WT-L and AT2-L groups had higher levels of TNF-α when compared to their control groups, WT-NL and AT2-NL (*p* < 0.001 and *p* < 0.01, respectively) (Figure 2). AT1 knockout mice submitted to ligature-induced periodontitis (AT1-L) had much lower levels of TNF-α in gingival tissue when compared to WT-L and AT2-L, both submitted to periodontitis (*p* < 0.001).

We performed the biochemical evaluation of the renin-angiotensin system through the levels of angiotensin II and angiotensin 1-7 in gingival tissue. Higher levels of angiotensin II and angiotensin 1-7 were found in the AT2-L group compared with its control group AT2-NL (*p* < 0.01 and *p* < 0.01, respectively; Figure 2). Figure 2 also shows that AT2-L had higher levels of angiotensin 1-7 in gingival tissue when compared to AT1-L (*p* < 0.05).

### 2.3. Gene Expression Analysis by RT-PCR

We analyzed the genic expression of Toll-like receptor 2 (TLR2) to evaluate the inflammatory response and also the genic expression of the following RAS components: angiotensin-converting enzyme (ACE), angiotensin 2 (ACE2) and Mas receptor (MasR).

**Figure 3:**
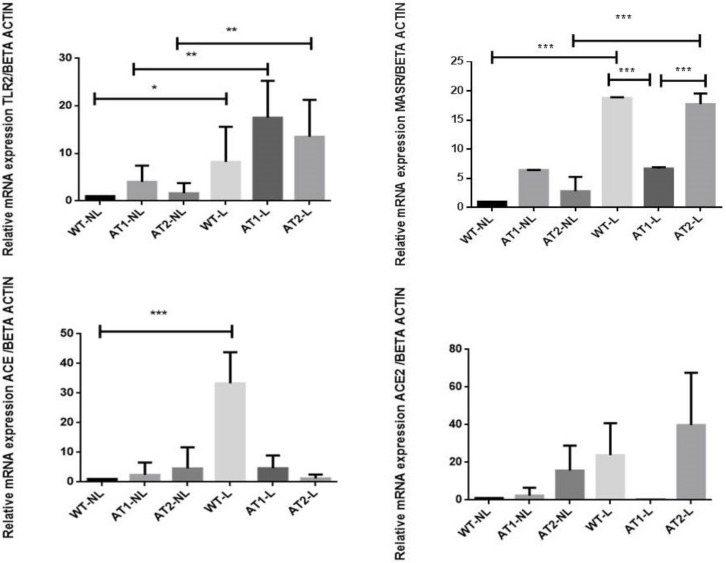
**Relative mRNA expression results for TLR2, MasR, ACE and ACE2 for the non-ligature and ligature groups of the three knockout mouse strains.** All graphs show mean ± SE. (standard deviation). Statistical differences by brackets labeled by * *p* < 0.05, ** *p* < 0.01, *** *p* < 0.001, comparing WT-NL vs. WT-L, AT1-NL vs. AT1-L, AT2-NL vs. AT2-L, WT-L vs. AT1-L, AT1-L vs. AT2-L. (Graph Pad Prism 6.01 software). *n* = 5 animal per group.

Figure 3 shows that ligature-induced periodontitis increased the TLR2 relative RNA expression as significant differences were found between the ligature groups (WT-L, AT1-L and AT2-L) and their respective control groups, without ligature, WT-NL (*p* < 0.05), AT1-NL (*p* < 0.01) and AT2-NL (*p* < 0.01).

The ligature-induced periodontitis significantly increased the Mas relative RNA expression in wide type mice (WT-L) and AT2 knockout mice (AT2-L), when compared to their control groups, WT-NL and AT2-NL (*p* < 0.001 and *p* < 0.001, respectively) (Figure 3), and in the AT1-L knockout mice, *p* < 0.001 (Figure 3).

The relative RNA expression of ACE was significantly higher in the wide type mice submitted to ligature-induced periodontitis (WT-L) when compared to its control group, WT-NL (*p* < 0.001), suggesting that the constitutive silencing of AT1 or AT2 receptors prevented the increase of ACE gene expression, induced by periodontitis.

No significant differences were found in the ACE2 relative RNA expression between the groups submitted to periodontitis and their control groups, without ligature (Figure 3).

## 3. Discussion

Scientific evidence shows that periodontal disease is clinically and subclinically associated with cardiovascular diseases in several populations and that patients with periodontal disease have an accelerated risk of developing cardiovascular diseases [20].

In this study, we evaluated the influence of the renin-angiotensin system on ligature-induced periodontitis, more specifically, the interaction between angiotensin II and its receptors, as several studies have reported the participation of this system in the pathogenesis of inflammatory disorders [12,21].

The renin-angiotensin system having been associated with bone remodeling in osteoporosis [22], in addition, Ang II has been identified as an essential pathophysiological activator of inflammatory mechanisms [15]. The classical RAS axis is ACE/Ang II/AT1 receptors, are responsible for vasoconstriction and also for pro-inflammatory effects [13]. Angiotensin II type 2 receptors (AT2), less expressed than type 1 receptors (AT1) [17], have functions that counteract the effects of the AT1 receptor [11].

Our study proposed to assess the role of AT1 and AT2 receptors in periodontitis, submitting AT1 or AT2 knockout mice to ligature-induced periodontitis.

The linear bone loss analysis showed that ligature was able to induce periodontitis, suggesting that the experimental model used in this study is suitable to study the pathogenesis of periodontitis.

Micro-computed tomography detected a greater bone loss in AT1 knockout mice when compared to AT2 knockout mice, and both groups were not subjected to periodontitis. In agreement, we have previously demonstrated that the constitutive silencing of AT1 in mice had a negative impact on bone. The constitutive blockade of AT1 altered bone density, the quality and number of bone trabeculae and decreased the number of osteoblastic cells [23]. The lower number of osteoblasts can partially explain the lower bone density observed in AT1 knockout animals. Akagi et al. [24] also noted that AT1 receptor deficiency did not significantly improve the erosive bone changes associated with arthritis. These findings, at first, are unexpected, because AT1 has been reported to be a negative regulator of bone remodeling [25], whereas AT2 is generally associated with bone protective effects [17]. Together, these findings suggest that AT1 is critical for bone homeostasis and regulates bone loss in inflammatory conditions and that the AT2 receptor signaling pathway seems to be regulated by the AT1 receptor.

The RAS in the wild-type groups is free to follow the natural course of its pathway, triggering regulatory mechanisms when necessary. In the present study, the AT1 blockade was shown to reduce the levels of proinflammatory cytokines, such as IL-1ß, IL-6 and TNF-α, but was not able to prevent the bone loss, a hallmark of periodontitis progression.

We found a significant increase in gene expression of the Mas receptor in the group of AT2 knockout mice submitted to periodontitis (AT2-L) compared to AT1-L, probably by a compensatory mechanism in response to AT2 blockade. In other words, we speculate that AT2 blockade justifies the greater amount of free Ang 1-7, found in AT2 knockout mice, but not in AT1 knockout animals. Our study also shows that periodontitis induced a significant increase in gingival levels of angiotensin II in AT2 knockout mice, but not in AT1 knockout animals. The increase in angiotensin 1-7 levels may be partially associated to the fact that angiotensin 1-7 acts via Mas and AT2 receptors, as previously discussed.

The Ang 1-7/MasR axis is reported to exert anti-inflammatory effects, unlike the common Ang II/AT1 axis [26] and is therefore a neutralizer of Angiotensin II actions [11].

In an investigation into the effects of Angiotensin 1-7 on joint inflammation and cardiac complications in an arthritis model, Wang et al. [27] demonstrated that Angiotensin 1-7-treated mice showed reduced TNF-α, IL-1β, and IL-6 levels, with reduced synovial tissue hyperplasia, inflammatory infiltrate, and bone destruction, demonstrating a protective effect of angiotensin 1-7 on joints. Ang 1-7 was also able to reduce the secretion of inflammatory cytokines. Despite the higher levels of Ang 1-7 and the higher gene expression of MasR in the AT2 knockout group, we did not find any statistical differences in the gene expression of ACE2, the enzyme responsible for the cleavage of Ang I into Ang II, among the groups.

In the present study, we observed a significant increase in TLR-2 gene expression in all groups of animals submitted to experimental periodontal disease. Toll-like receptors are pattern recognition receptors which play a crucial in the initiation of innate immune response by detecting potential harmful pathogens. TLR2 transduces in particular gram-positive ligand signals [28]. Responses to Porphyromonas gingivalis, for example, are predominantly mediated by this receptor, stimulating the production of inflammatory cytokines, chemokines and antimicrobial peptides [29]. Furthermore, it was observed that the expression of TLR2 is greater near the epithelium of periodontal pockets than in the gingiva, in humans [28].

Experimental animal models are critical tools for investigating the mechanisms of periodontal pathogenesis. Rodents are relevant for experimental periodontal research, as they present a structure of the gingival dental region that is very similar to that observed in humans. An important advantage of the ligature-induced periodontitis model selected for the present study is that the disease can be initiated at a known time with a predictable sequence of events culminating in alveolar bone loss within a few days. The removal of ligatures allows an investigation of the resolution of inflammation and the healing response [30]. The technical procedure involves placing ligatures around a posterior tooth. Ligatures are believed to facilitate local bacterial accumulation and thus increase bacterial-mediated inflammation and bone loss [31,32]. The major limitation of the ligature-induced periodontitis in mice is due to the technical challenges related to the relatively small size of the oral cavity of these animals.

The use of knockout animals for AT1 and AT2 receptors is another strong point of our work, as it is a promising approach in understanding the pathophysiology of diseases in addition to avoiding adverse effects related to the pharmacological inhibition. However, the constitutive lack of the gene encoding the AT1 or AT2 receptors can induce compensatory mechanisms that should be considered and discussed.

The use of high-resolution microcomputed tomography to assess alveolar bone loss is another strength of this study. Micro Ct provides 3D information and allows a more representative analysis in the whole sample extension. Bone microarchitecture analysis showed a significant reduction in bone tissue volume (BV/TV%) in AT1 knockout animals without ligature. After ligature, all animals including the control wide-type group, showed significant linear bone loss, confirming that ligature was able to induce bone loss. The ratio of bone volume (BV) to total volume (TV) (BV/TV%) showed that AT1 knockout mice not subjected to ligature-induced periodontitis had a significant lower bone volume when compared to AT2 knockout mice without a ligature. These findings suggest a possible role for the AT1 receptor in the regulation of bone metabolism, possibly regulating AT2 signaling.

**Figure 4:**
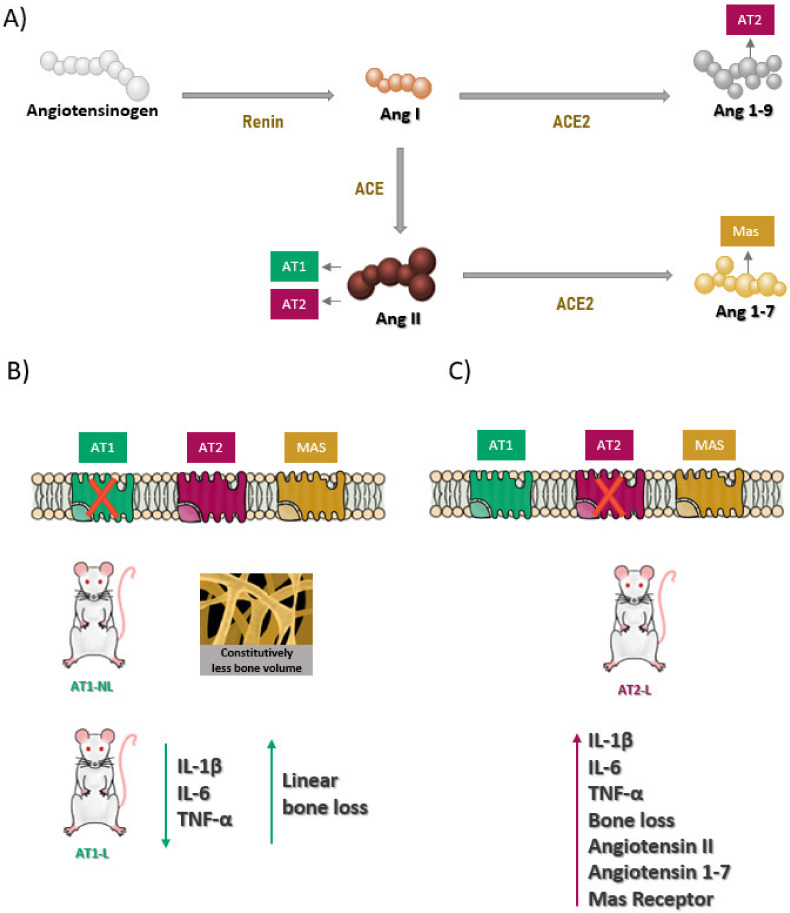
(**A**) Pathways of the renin-angiotensin system in the wild-type animal; (**B**) free pathways of the renin-angiotensin system for AT1 receptor knockout animals and changes in cytokine levels after induction of periodontitis by ligation; (**C**) free pathways of the renin angiotensin system for AT2 receptor knockout animals and changes in cytokine levels and in RAS (Angiotensin II and Angiotensin 1-7), Mas receptor after induction of periodontitis by ligature.

Molecular characteristics after periodontitis induction showed the following signaling pathways: WT-L: significant increase in signaling pathways for IL-1 beta, IL-6, IL-10, TNF-α and Mas receptor; AT1-L: significant reduction in IL-1 beta compared to WT-L; AT1-L: significant reduction in IL-6 and TNF-α significant reduction compared to WT-L and AT2-L; AT1-L: significant reduction in Angiotensin 1-7 compared to AT2-L; AT2-L: significant increase in IL-1 beta, IL-6, TNF-α, Angiotensin1-7 and Angiotensin II compared to its AT2-NL control (Figure 4).

## 4. Material and Methods

### 4.1. Animals

The experimental protocol followed the ARRIVE guidelines for animal research suggested by the National Center for the Replacement, Refinement, and Reduction for Animals in Research. FVB/N mice, AT1 receptor Knockout mice, and AT2 receptor Knockout mice were kept in a risk vivarium I (UFRN Biophysics and Pharmacology Experimental Experiment Laboratory-LAFINC) in a ventilated rack with mini-insulators at 22 °C, 12 h light/dark cycle, with an exhaust having a capacity of 20 air exchanges/hour. The different mice strains were obtained of the animal house of genetically modified rodents produced at the Max Delbruck Center for Molecular Medicine (Berlin, Germany). The knockout animals were donated by Professor Robson Santos from the Federal University of Minas Gerais (Belo Horizonte, Brazil). All animal protocols were approved by the Animal Ethics Committee of the Federal University of Rio Grande do Norte (CEUA, Protocol No. 046/2017, 9 April 2019), Brazil.

### 4.2. Study Groups

There were 2 study groups for wild FVB/N mice: Wild-Type (WT-NL)-non-ligature of periodontitis experimental model (15 animals, male), and Wild-Type ligature (WT-L) of periodontitis experimental model (15 animals, male).

There were 2 study groups for the AT1 receptor knockout group: AT1 Healthy (AT1-NL) non-ligature of periodontitis experimental model (15 animals, male) and AT1 receptor knockout ligature (AT1-L) of periodontitis experimental model (15 animals, male).

There were 2 study groups for the AT2 receptor knockout group: Healthy AT2 (AT2-NL)—non-ligation of the experimental periodontitis model (15 animals, male), and AT2 receptor knockout ligature (AT2-L) of periodontitis experimental model (15 animals, male).

A power calculation was performed to determine the sample size. The sample size was determined to provide 80% power to recognize a significant difference of 20% among groups and the standard deviation of 15% with a 95% confidence interval, considering the linear bone loss. Therefore, a sample size of 5 animals/analysis was required.

### 4.3. Induction of Ligature Periodontitis Experimental Model

**Figure 5:**
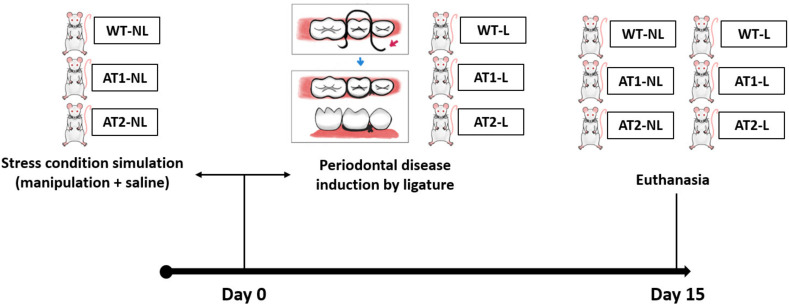
Design study. Wild-Type-non-ligature of periodontitis experimental model (WT-NL) and Wild-Type ligature of periodontitis experimental model (WT-L). AT1 Healthy non-ligature of periodontitis experimental model (AT1-NL) and AT1 receptor knockout ligature of periodontitis experimental model (AT1-L). AT2-non-ligature of the experimental periodontitis model (AT2-NL), and AT2 receptor knockout ligature (AT2-L) of periodontitis experimental model. Induction of periodontitis was performed in experimental groups (WT-L, AT1-L or AT2-L) by placing a 5.0 nylon thread on the mouse left second molar. Euthanasia was performed on day 15, after the gingival and maxillary tissue samples were sent for analysis.

Induction of periodontitis was performed in experimental groups (WT-L, AT1-L or AT2-L) by placing a 5.0 nylon thread on the mouse left second molar under intraperitoneal injection (i.p) ketamine (80 mg/kg) and xylazine (10 mg/kg) anesthesia. Thiopental (80 mg/kg) euthanasia was performed on day 15. Afterwards, gingival and maxillary tissue samples were sent for analysis. The sequence of the study design is showed in Figure 5.

### 4.4. Micro-CT

The Maxilla (WT-NL or L, AT1-NL or L, AT2-NL or L) were fixed in 10% formaldehyde for 48 h and then stored in 70% alcohol. The maxilla was scanned using a micro computed tomography (micro-CT) scanner (Skyscan 1172; Skyscan, Kontich, Belgium) with a voxel size of 10 μm (isotropic voxel) and X-ray energy of 55 KVp and 181 μA. Each scan was performed over a period of 21 min with 0.4° rotation. Ten-frame averages were taken and a 0.5 mm aluminum filter was used. Virtual image slices were reconstructed using the tapered beam reconstruction version 1.5 software program based on the Feldkamp algorithm.

**Figure 6:**
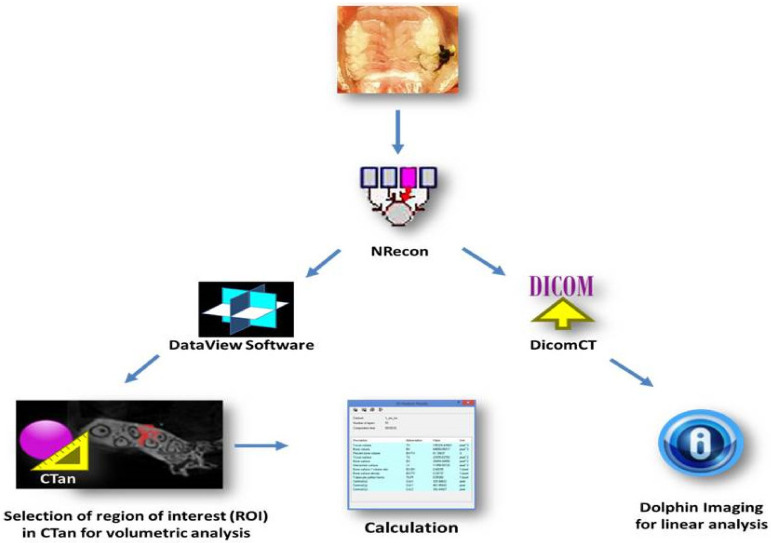
Scanner sequence of maxilla and linear and volumetric analysis by the software programs.

The scan images acquired by micro-CT was imported to a computer and rebuilt using the Nrecom software program (version 1.6.10.1, Skyscan, Bruker, Belgium) in approximately 75 slices. The scan images rebuilt by Nrecom software were oriented using DataViewer (V.1.5.2 Bruker, Billerica, MA, USA) to assess volumetric bone levels. Next, volumetric measurements (BV/TV %) were taken from the maxilla (slice where the roots of the first to the last molar can be seen, 15 slices below that slice, and 50 slices above that slice) using CTAn (CT analyser Version 1.13.11.0, Bruker, Belgium), (03 slices-region of interest/ROI) (Figure 6).

Micro-computed tomography (Maxilla) data were imported in DICOM (DicomCT version 2.1, Skyscan, Bruker, Belgium) format into the Dolphin^®^ software package (Dolphin Imaging, Chatsworth, CA, USA) (Figure 6) (Linear analysis). The middle of the crown in the axial plane was identified and linear bone distances (mm) were recorded at the mesial and distal 2nd molar on the sagittal image and at the palatal and buccal 2nd molar on the coronal sagittal image. Linear measurements were recorded from the cementum enamel junction (CEJ) to the alveolar bone crest (ABC). Each 2nd molar received 04 measurements calculated for each group in mm. This step was performed at UCLA in the United States through an international collaboration with UCLA periodontics.

### 4.5. Elisa Immunoassay for Detecting IL-1β, IL-6, IL-10, TNF-α and Peptides

Gingival tissues (*n* = 5) from the groups (WT-NL or L, AT1-NL or L, AT2-NL or L) were stored at −70 °C. IL-1β levels (detection range: 62.5–4000 pg/mL; lower detection limit: 12.5 ng/mL recombinant mouse IL-1β), IL-6 (detection range: 125–8000 pg/mL; lower detection limit: 50 ng/mL recombinant mouse IL-6); IL-10 levels (detection range: 31.2.5–2000 pg/mL; lower detection limit: 14.7 ng/mL recombinant mouse IL-10); TNF-α (detection range: 62.5–4000 pg/mL; lower detection limit: 50 ng/mL recombinant mouse TNF-α); Angiotensin II (detection range: 35.25–2000 pg/mL) and angiotensin 1-7 (detection range: 7.813–500 pg/mL) were determined using commercial ELISA kits (R&D Systems, Minneapolis, MN, USA).

First, microtiter plates were coated overnight at 4 °C with mouse antibodies against IL-1β, IL-6, TNF-α, Angiotensin II and angiotensin 1-7. The plates were then blocked, samples and standards added in various dilutions in duplicate, and incubated at 4 °C for 24 h. The plates were washed three times with buffer and the antibodies added to the wells (anti-IL-1β, anti-IL-6, anti-IL-10, anti-TNF-α, anti-angiotensin and anti-angiotensin 1-7), biotinylated sheep polypropylene, diluted 1000 with 1% BSA assay buffer). The plates were incubated at room temperature for 1 h, washed, and 50 µL of avidin-HRP (1 uL:200 uL) was added. O-phenylenediamine reagent staining (50 mL) was then added 15 min later, and the plates were incubated in the dark at 37 °C for 15–20 min. The enzyme reaction was reduced with H_2_SO_4_ and absorbance measured at 490 nm. The values were expressed as pg/mL.

### 4.6. RT-PCR Gene Marker Analysis

Control (WT-NL, AT1-NL and AT2-NL) and experimental (WT-L, AT1-L and AT2-L) groups were included in the expression quantification by RT-PCR. Total RNA from gingival tissues of the treated groups was extracted using Trizol reagent (Invitrogen, Carlsbad, CA, USA) according to the manufacturer’s guidelines and stored at −70 °C.

RNA concentration was determined from the optical density at a wavelength of 260 nm (using an OD260 unit equivalent to 40 μg/mL of RNA). Five micrograms of isolated total RNA was transcribed to cDNA in a reaction mixture containing 4 μL 5× reaction buffer, 2 μL dNTP mixture (10 mM), 20 units of RNase inhibitor, 200 units of avian myeloblastosis virus (AMV) reverse transcriptase, and 0.5 μg oligo (dT) primer (High-Capacity cDNA Reverse Transcription Kit, Foster City, CA, USA) in a total volume of 20 μL. The reaction mixture was incubated at 42 °C for 60 min, and the reaction was terminated by heating at 70 °C for 10 min. The cDNA was stored at −80 °C until further use. Gene expression was assessed by PCR amplification using primer pairs based on published mouse sequences: Beta actin, TLR-2, ACE, ACE2, MASR (Table 1).

Quantitative RT-PCR was performed using Power SYBR Green master mix (Applied Biosystems, Waltham, MA, USA), and a Step One Plus thermal cycler (Applied Biosystems), according to the manufacturer’s instructions. Next, 2.5 μL of each cDNA was added in a final volume of 20 μL for the 1× PCR master mix. The PCR conditions were as follows: 95 °C for 5 min, 40 cycles of 30 s at 95 °C, 30 s at 52–60 °C (target-based), and 60 s at 72 °C. The relative quantitative fold change relative to the control (WT-NL) was calculated using the comparative Ct method, where Ct is the cycle number in which the fluorescence first exceeds the threshold. The Ct values for each sample were obtained by subtracting the values for GADPH Beta-Actin Ct from the Ct value of the target gene. The specificity of the resulting PCR products was confirmed by curve melting.

### 4.7. Statistical Analysis

Data were analyzed using descriptive (mean and standard deviation) and analytical statistics using parametric tests such as ANOVA, followed by a Bonferroni post-test and non-parametric Kruskal–Wallis test at a 5% significance level (Graph Pad Prism 6.01 software).

## 5. Conclusions

Our data suggest that the receptor AT1 appears to be important for the maintenance of bone mass, since silencing this receptor results in bone loss, even in healthy animals. AT2 receptor function in periodontal bone loss appears to be regulated by AT1, as increased bone resorption was observed in AT2 knockout mice, although AT2 has been associated with protective effects on bone. We believe that the AT2 receptor has a dual role on bone, depending on the crosstalk between AT1 and AT2 receptors. Further studies are needed to confirm this hypothesis.

## Figures and Tables

**Table 1:** Gene sequence forward and reverse (Beta actin, MasR, TLR-2, ACE2, ACE).

Primer	Forward	Reverse	Specie
**Beta-Actin**	AGGCCAACCTGTAAAAGATG	TGTGGTACGAGAGGCATAC	Mouse
**MASR**	AGAAATCCCTTCACGGTCTACA	GTCACCGATAATGTCACGATTGT	Mouse
**TLR2**	GCAGCCACTCTACCTGAACC	GCGCCACCAAATCATAGATGTTG	Mouse
**ACE2**	TCCAGACTCCGATCATCAAGC	TGCTCATGGTGTTCAGAATTGT	Mouse
**TLR-2**	CACCACTGCCCGTAGATGAA	GCCTGCGAATGCCAGCTT	Mouse

## Data Availability

Data supporting reported results can be found in Department of Biophysical and Pharmacology, Federal University of Rio Grande Norte, Natal, RN, Brazil.
